# Effects of bogus genetic feedback on subjective and physiological responses to acute stress

**DOI:** 10.1016/j.cpnec.2026.100358

**Published:** 2026-06-25

**Authors:** Susanne Vogel, Marie-Christin Barthel, Kim Lars Fricke, Markus Muehlhan, Nina Alexander

**Affiliations:** a Department of Psychology, Faculty of Human Sciences, MSH Medical School Hamburg, Am Kaiserkai 1, Hamburg, 20457, Germany; b ICAN Institute for Cognitive and Affective Neuroscience, MSH Medical School Hamburg, Am Kaiserkai 1, Hamburg, 20457, Germany; c Department of Psychiatry and Psychotherapy, Philipps University Marburg, Marburg, Germany; d Center for Mind, Brain and Behavior, Philipps University Marburg, Marburg, Germany

## Abstract

Recent advances in direct-to-consumer genetic testing have made individualized genetic risk feedback increasingly accessible, yet its psychological impact remains unclear. Initial findings suggest potential adverse outcomes, including increased worry and biased symptom reporting. Given that stress reactivity is partially heritable and a key risk factor for numerous stress-related conditions, understanding the influence of genetic feedback on stress reactivity is critical. In this study, we investigated how receiving bogus genetic feedback about stress vulnerability influences subjective, physiological, and cognitive responses to acute stress in 76 healthy participants undergoing the Trier Social Stress Test. Results confirmed successful participant deception and effective stress induction, as evidenced by subjective, endocrine, and cardiovascular stress markers. Participants informed of a genetic predisposition for high stress reactivity were more concerned and exhibited stronger restlessness directly after the negative feedback and following the stressor compared to control groups without this feedback. These results were specific to the stress vulnerability genetic feedback and were not observed in participants informed of an increased genetic risk for diabetes. In contrast, physiological stress responses and cognitive changes were not significantly affected. These findings demonstrate that genetic feedback alone can immediately induce concern and alter subjective responses to stress without corresponding physiological changes, suggesting that stress experience may be particularly prone to expectation effects following genetic risk information. Our results underscore the need for careful consideration of how genetic risk communication may shape stress-related health outcomes, and future research should explore the long-term consequences of genetic risk feedback.

## Highlights

• Healthy adults received bogus genetic feedback regarding their stress susceptibility.
• Perceived genetic risk increased concern and subjective stress reactivity.
• Physiological stress markers were not significantly affected by genetic feedback.
• Working memory was not significantly affected by genetic feedback.

## 1 Introduction

Recent years have seen an impressive rise of direct-to-consumer (DTC) genetic testing such that millions of customers have received personalized genetic information [1]. These tests allow individuals not only to search for unknown relatives and ancestors, but also to obtain health-related information. Since human traits and complex diseases are partly heritable [2] and an increasing number of risk variants have been identified in genome-wide association studies (GWAS), DTC genetic tests can be used to derive polygenic risk scores for thousands of conditions [3].

Young adults, in particular, appear highly interested in learning about their individual genetic risk, e.g., for stress-related conditions such as major depressive disorder. However, knowledge about how to interpret such information is often limited [4]. As genetic feedback obtained through DTC testing is usually not accompanied by expert counseling or medical guidance, understanding its psychological consequences is of increasing importance. Although expectations are high that genetic risk information may support informed healthcare and lifestyle decisions [5], the consequences of receiving such feedback remain complex and insufficiently understood.

Several initial studies have demonstrated profound psychological effects of genetic risk feedback. Receiving information about an elevated genetic risk for adverse health outcomes has been associated with negative emotional reactions, increased worry, and a heightened sense of genetic determinism, which may reduce perceived self-control or confidence in treatment effectiveness [[6], [7], [8], [9]]. Moreover, genetic risk communication can influence cognition and self-perception in line with the expectations raised by the information. For example, participants informed about carrying a risk variant for Alzheimer's disease performed worse in memory tests than individuals with the same risk variant who were unaware of their status [10]. This detriment was accompanied by a long-term increase in test anxiety and lower self-rated memory capabilities. Likewise, genetic feedback regarding depression has been shown to increase retrospective symptom reporting and to reduce confidence in emotion regulation abilities [11], [12]. These studies suggest that genetic risk information may induce labeling effects similar to nocebo responses, whereby expectations negatively affect psychological outcomes [13]. Importantly, such effects may have clinical relevance, as diagnostic assessments often rely on self-reported symptoms [14]. Other studies, however, have reported no substantial effects of genetic feedback on distress, health, or behavioral change (e.g., Ref. [15]). Overall, current evidence suggests that genetic risk feedback can influence emotional and cognitive processes in line with the expectation raised by the genetic information, although findings remain heterogeneous.

Research on placebo and nocebo effects further indicates that expectation effects can extend to physiological outcomes [16]. Consistent with this notion, a pioneering study demonstrated that participants who believed to carry a genetic risk for obesity showed physiological responses congruent with the risk communicated (e.g., cardiorespiratory physiology during exercise, [17]). Notably, the physiological effect of believing to carry a genetic risk was in some cases greater than the effect of the actual genetic risk. These findings highlight the importance of understanding both psychological and physiological consequences of genetic risk communication.

In this regard, induced stress responses are a particularly suitable model for testing whether beliefs about biological vulnerability can shape stress experience and stress-related physiology. Stress is a major contributor to the onset and maintenance of numerous mental and somatic conditions which place a great burden on individuals and society [18]. According to transactional stress models, stress responses arise when individuals appraise situational demands as exceeding their coping resources [19]. Stress then comprises subjective experiences as well as cognitive and physiological components that jointly support adaptation to challenging situations [20], yet individuals differ strikingly in the strength of their responses, i.e., their stress reactivity. Importantly, dysregulation of central stress systems, including the hypothalamus-pituitary-adrenal (HPA) axis and the autonomic nervous system (ANS), has been implicated in various clinical conditions [21]; [22].

In recent years, several genetic variants have been associated with individual differences in physiological stress reactivity, including cardiovascular and endocrine responses to stressors (e.g., Ref. [[23], [24], [25]]). Although findings have not always been consistent and effect sizes are typically small, commercial DTC tests claim to provide information about genetic susceptibility to stress. Given that stress experience depends strongly on appraisal and perceived coping ability, genetic feedback suggesting increased stress sensitivity may induce expectation-congruent psychological and physiological responses. However, it remains unclear whether merely believing to carry a genetic susceptibility to stress is sufficient to alter acute stress responses.

The primary aim of the present study was to investigate whether a believed high vs. low genetic susceptibility to stress affects subjective stress reactivity and physiological responses to a socio-evaluative stressor. Therefore, we compared three groups and hypothesized that participants informed about a genetic predisposition for heightened stress reactivity would show stronger subjective and physiological responses to the Trier Social Stress Test (TSST; [26]) compared to both, a control group receiving low-risk genetic feedback, and a “bad news” control group receiving increased genetic risk feedback for type 2 diabetes, to examine specificity of potential effects. As secondary aims, we tested whether the genetic risk information influences retrospective reports of chronic stress in line with a labeling effect and whether previously reported changes in working memory (WM) performance in the context of stress [27]; [28] are exacerbated by a believed high genetic susceptibility to stress.

## 2 Methods & materials

This study was preregistered on OSF before data acquisition was completed and any data analysis was conducted (https://osf.io/wymv7).

### 2.1 Participants and sample size

Our preregistered target sample size was 24 participants per group (N = 72) with a balanced distribution of (self-identified) men and women per group (non-binary individuals did not sign up). This sample size was supported by an a-priori power calculation for our first hypothesis, as it allows for the detection of medium-sized within-between interaction effects (*f* = .25, 3 groups, 6 repetitions of stress measurements) using an α-level of .05 and default values for correlation among repeated measures and non-sphericity correction with an estimated power of 99% (G∗Power 3.1.9.2; [29]). Please note, however, that testing for interactive effects of gender and group would have resulted in small cell sizes and reduced power, particularly for small effects, and was thus omitted. Due to freezer malfunctioning, saliva samples were lost for four participants. As cortisol reactivity was one of our main outcome measures, we recruited four additional participants, resulting in a final N = 76.

Participants were recruited through advertisements on campus and online using the local student participation system. Eligibility for participation was established in a telephone screening, during which we also obtained information on potential covariates (smoking status as self-reported smoker or non-smoker, oral contraceptive use). Participants were between 18 and 35 years of age and fluent in German. As the menstrual cycle can affect the cortisol system [30], women had to use hormonal contraceptives or have a regular cycle, in the latter case the stress induction was scheduled in the luteal phase. It should be noted that participants taking hormonal contraceptives typically show a reduced cortisol stress response compared to participants in the luteal cycle phase [31], whereas findings regarding effects on autonomic stress reactivity are inconsistent [32]. We therefore included hormonal contraceptive use as a covariate rather than excluding these participants, in order to retain generalizability. Exclusion criteria were any (history of) self-reported physical, psychiatric, neurological, endocrine, cardiovascular, internal, or metabolic diseases, medication intake that would affect stress reactivity (except for hormonal contraceptives), glaucoma, extreme weight (rounded body-mass-index [BMI], <18 or >30), vaccination in the past month, pregnancy or nursing, excessive smoking or alcohol intake, and use of illicit drugs. In contrast to our preregistration, we decided to include a) individuals with irregular (non-daily) use of cannabinoids, b) proportionally more female participants, and c) an individual taking topical (non-steroid) medication (skin cream) for skin problems to ensure completion of data collection within the planned time frame. Participants were reimbursed for participation by partial course credit.

### 2.2 Study design, procedure, and stress reactivity assessment

**Fig. 1:**
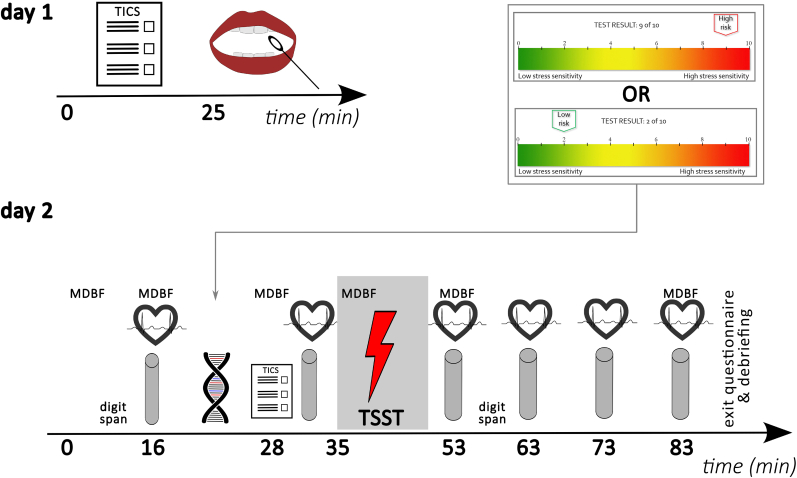
**Experimental procedure on both testing days.** On day 1 (top), participants provided informed consent, answered a questionnaire (Trier Inventory for the Assessment of Chronic Stress, *TICS*) and provided a buccal sample for pretend DNA analysis. On day 2 (bottom), participants returned to the laboratory, provided initial subjective stress measures (*MDBF*), performed a working memory task (*digit span*), followed by a baseline saliva sample for cortisol analysis (*cylindric icon*) and vital signs measurement (*heart icon*) for physiological stress assessment. They then received bogus genetic risk feedback (indicated by the *DNA symbol*) as illustrated in the inlet (top right) according to group assignment. Please note that the actual feedback in the study was provided in German and an additional condition was included informing participants to be at risk for type 2 diabetes (see main text and supplement). Afterwards, they answered the TICS screening scale and all stress markers were assessed again. Participants then underwent the Trier Social Stress Test (*TSST*) in a separate room and continued providing stress assessments until the end of the experiment. A second working memory assessment occurred between saliva samples 3 and 4. Finally, they answered an exit questionnaire regarding the genetic feedback manipulation to test successful deception. Time is indicated in minutes.

To investigate the subjective and physiological effects of (bogus) genetic feedback, we used a three-group between-subjects design (Fig. 1). On Day 1, all participants provided written informed consent to enroll in a study purportedly investigating the “genetic underpinnings of individual differences in cortisol stress reactivity”. Next, they answered a questionnaire regarding stressful experiences in the past three months to assess chronic stress levels (Trier Inventory for the Assessment of Chronic Stress [TICS]; [33]) and depressive symptoms in the past two weeks (German version of the Beck Depression Inventory II [BDI-II]; [34]). Participants with a BDI-II score of 20 or higher were excluded from continuing with the experiment and debriefed to adhere to local ethics regulations. Afterwards, participants provided a buccal sample for pretend genetic analyses by brushing a cotton swab along the inner side of the mouth. Finally, all participants were told not to eat, do sport, or drink anything but water for 2 h prior to their second appointment. After Day 1, participants were pseudo-randomly assigned to one of three experimental groups while balancing for (self-reported) gender.

On Day 2 (3 to 10 days after day 1, except for a 2-days delay in n = 1 due to scheduling issues), participants returned to the laboratory. They first answered a questionnaire to assess their subjective stress levels (Mehrdimensionaler Befindlichkeitsfragebogen, MDBF, see below for more details; [35]), followed by an assessment of WM capacity (see below). Baseline vital signs (heart rate, blood pressure) were assessed using automatic wrist blood pressure monitors (Omron, the Netherlands) to index ANS activity; vitals were taken twice and averaged to improve robustness. Participants also provided a baseline saliva sample to assess HPA activity, i.e., cortisol concentrations, using Salivettes “code blue” (Sarstedt®, Germany) and completed a second subjective stress assessment. Participants then received (bogus) information on their individual genetic predisposition for either heightened stress sensitivity (group 1), low stress sensitivity (group 2), or low stress sensitivity but an increased risk for type 2 diabetes (group 3). This feedback was provided orally and accompanied by a written report (see Supplementary Material) explaining the test rationale, the single nucleotide polymorphisms (SNPs) which were supposedly investigated, the individual purported genotype per SNP, the construction of a polygenic risk score, a visual risk scale with the personalized result highlighted (Fig. 1), a short interpretation of the result, and a printout of the (simulated) DNA test (sequencing chromatogram) from the laboratory. Importantly, the feedback differed between groups. In group 1, the last part read *“The result of the DNA test indicates a high risk of being very susceptible to stress. This means, for instance, that carriers of this genetic risk profile release more stress hormones and show a greater heart rate increase during stress.”* For group 2, the feedback was reversed, i.e., indicating a low risk. For group 3, an additional paragraph regarding *“other test results”* was added below the low-risk feedback: *“The result of the DNA test indicates a high risk of type II diabetes mellitus, so-called “adult-onset diabetes”. A high risk of type II diabetes leads to increased blood glucose levels due to a steadily developing insulin resistance. This means that you should follow a specific (*e.g.*, low-sugar) diet, especially as you get older.”*

Afterwards, subjective and physiological stress markers were assessed again, and participants answered a subscale of the TICS (i.e., the screening scale) to assess potential effects of genetic feedback on retrospective chronic stress reports without repeating the test verbatim. Participants then underwent the TSST ([26]; see below) in a separate room, including a subjective stress assessment during the preparation phase. After the TSST, participants were brought back to the initial test room and another assessment of subjective and physiological stress measures and WM followed. Physiological stress assessments were repeated every 10 min until 50 min after stress onset. Participants then completed a final subjective stress assessment and a short exit questionnaire regarding the genetic feedback manipulation. In this questionnaire, participants indicated whether they believed that a buccal sample had been taken to analyze their DNA regarding their biological stress reactivity, whether they believed that they had a high genetic sensitivity to stress, had a high genetic risk for diabetes, and whether they were concerned about the result of their DNA test (1 = don't agree at all, 5 = strongly agree). Finally, they were fully debriefed as to the study's true purpose and informed about the possibility to retract their data. All TSSTs were conducted between 1 and 6 p.m. to control for diurnal variation in cortisol secretion. The study was approved by the local ethics committee (Ethikkommission der Medical School Hamburg, MSH-2019/75). All methods were performed in accordance with relevant guidelines, regulations and the current version of the Declaration of Helsinki.

### 2.3 Trier Social Stress Test

The TSST is considered as gold-standard for psychosocial laboratory stress induction in humans leading to robust subjective, endocrine, and autonomic stress responses by combining socio-evaluative threat and unpredictability [36]. More precisely, it simulated a 15-min job interview and consisted of a 5-min preparation phase (including subjective stress assessment), a 5-min speech about the participant's personal eligibility for his/her favorite job, and a 5-min mental-arithmetic task (counting backwards from 2043 in steps of 17). During the entire 15 min, participants were videotaped and evaluated by two neutral, non-reinforcing committee members (one man and one woman), dressed in laboratory coats.

### 2.4 Salivary cortisol analyses

Saliva samples were frozen and stored at −20°C after each participant's test session and subsequently transferred to −80°C before shipping to the laboratory upon study completion. After thawing, salivettes® were centrifuged at 3000 rpm for 5 min, which resulted in a clear supernatant of low viscosity. Salivary concentrations were measured using commercially available chemiluminescence immunoassay with high sensitivity (IBL International, Hamburg, Germany). The intra- and interassay coefficients for cortisol were both below 7%. In addition to the four participants whose cortisol data were lost due to freezer malfunctioning, two participants had insufficient saliva at all time points and were excluded from cortisol analyses. Another six participants had partially missing cortisol data at singular time points.

### 2.5 Assessment of subjective stress reactivity

To assess effects of acute stress and genetic feedback on subjective stress, we used the MDBF (short form A). It assesses three dimensions (low vs. elevated mood, restlessness vs. calmness, and sleepiness vs. wakefulness), with four items each (1 = not at all, 5 = very much). Sum scores per scale can range from 4 to 20; high scores represent elevated mood, calmness, and wakefulness, respectively. The MDBF is frequently used in laboratory stress research using human samples in German-speaking countries as a way to measure subjective effects of stress (e.g., Ref. [[37], [38], [39], [40]]), typically demonstrating increased restlessness and lower mood after stress induction.

### 2.6 Assessment of chronic stress

The TICS was used to assess chronic stress experienced in the past 3 months [33] and to examine whether this retrospective evaluation might be affected by genetic feedback. On day 1, the full version consisting of ten scales was administered, assessing the frequency of stressful experiences related to, for instance, work overload, social overload, pressure to perform, or chronic worrying (0 = never, 4 = very often). Twelve of the 57 items form a screening scale providing an approximate index of chronic stress. To minimize memory effects, chronic stress assessment on Day 2 was limited to the TICS screening scale (TCSS).

### 2.7 Working memory assessment

To test changes in WM capacity from before to after the TSST, we used digit span tests [41]. Both forward and backward span were assessed orally by the experimenter using two trials per digit length in each condition. In line with the original instructions, the experimenter always started with the shortest digit span of the forward condition (3 digits, e.g., 4–6–2) and continued with increasingly longer spans until the participant failed to correctly recall both trials of a given length. Directly thereafter, the backward condition was administered in which participants had to repeat digit sequences in reverse order, starting again with the shortest span length (2 digits). For the first test, the original items from the manual were used. For the second test, a different set of self-developed items was used to avoid item-specific memory or carryover effects. Several outcome variables of the digit span task have been reported in the literature. We analyzed the absolute digit span (longest correct span), the total number of correct trials, and the product of these as an integrative measure of working memory capacity [42]. It should be noted, though, that due to the lack of a non-stressful control condition, potential differences in WM capacity from before to after the TSST cannot be unequivocally attributed to the TSST, as they may reflect both stress induction and the mere passage of time.

### 2.8 Handling of missing data

We replaced missing values in stress-reactivity outcomes (one MDBF item in one participant and missing cortisol data in six participants with partial data loss) by multiple imputation, creating five imputed datasets. In order to do so, we used all individuals who finished the study and all variables in the dataset, preset the initialization number to achieve reproducibility (Mersenne twister, initialization value: 2,000,000) and used the automatic procedure implemented in IBM SPSS Statistics 27 across all groups. Moreover, for the imputation of one missing MDBF item (n = 1), we defined possible minimum and maximum values and rounded the imputations to the nearest integer to retain its categorical nature. Other variables with missing data which were not of interest to the current analyses (e.g., participant's study year) were used only as predictors in the model. Please note that graphs and tables illustrate original data (before imputation).

### 2.9 Data analysis

We used *p* < .05 as criterion for significance. Stress reactivity was quantified using pre-post changes (baseline to peak) for subjective stress, blood pressure, and heart rate as descriptive summary measures. For cortisol data, we computed the area under the curve with respect to the increase (AUCi) and the ground (AUCg), respectively ([43]).

Cortisol levels for all time points and AUC indices deviated significantly from normal distribution (indicated by significant Shapiro-Wilk tests: all *p* < .05) and were log10-transformed. As AUCi contained negative values, the minimum value was added prior to log transformation. Transformation improved normal distribution of individual cortisol values and AUCg (*p* > .05), only AUCi remained non-normally distributed; therefore, untransformed AUCi values were retained for analyses. Outliers were defined for all metric measures as values deviating more than 3 SD from the mean and excluded from the respective analysis involving this variable (at most n = 2 per measure).

We then determined whether potential known covariates (i.e., gender, age, smoking status, and oral contraception [yes, no]) were associated with the cortisol response to the TSST (i.e., lgAUCg and AUCi) using independent samples *t*-tests and a Pearson correlation. Neither AUCi nor AUCg were significantly affected by any of these variables (AUCi: all *p* ≥ .090, lgAUCg: all *p* ≥ .057). As none of these variables differed significantly between groups (Fisher's exact tests and ANOVAs, respectively, all *p* ≥ .6), they were not included as covariates.

Although not preregistered, we included a manipulation check regarding successful deception before turning to our hypotheses. More precisely, we used ANOVAs to test whether the three experimental groups differed in their responses to the exit questionnaire. Post-hoc LSD tests were used in case of a significant effect of group.

To test our main hypothesis, we used repeated-measures (rm)ANOVAs with the between-subjects factor group (three genetic feedback groups) and the within-subjects factor time (six levels) to analyze the effects of (bogus) genetic feedback on stress reactivity, separately for the three MDBF-scales (indicating subjective stress), cortisol levels, heart rate, and blood pressure. Greenhouse-Geisser correction was applied when sphericity was violated. Baseline cortisol was included as a covariate in analyses on aggregate cortisol data (lgAUCg). If group showed a significant interaction with time, group differences were examined at each time point using post hoc pairwise t-tests.

To test our second hypothesis that genetic feedback might induce a recall bias on reported chronic stress levels, we used another rmANOVA on the TCSS data with the between-subjects factor group (three levels) and the within-subjects factor time (two levels, i.e., Day 1 and after genetic information). Finally, rmANOVAs with the between-subjects factor group (three levels) and the within-subjects factor time (two levels) were used to assess whether changes in WM capacity from before to after the TSST were affected by genetic feedback. Repeated measures ANOVAs were performed on the original dataset to avoid problems pooling the inference statistics, whereas analyses conducted on the individual imputed datasets yielded highly similar results. For other tests (correlations, t-tests), pooled statistics are reported. Analyses were conducted using IBM SPSS Statistics 27 and the package *MKmisc* for t-tests using multiple imputation in R 4.2.1 and R studio 2023.09.1.

## 3 Results

### 3.1 Sample description

In total, 88 individuals enrolled in this study. Of these participants, 11 did not complete the study due to termination of the TSST by the participant (n = 5), exclusion based on BDI-score (n = 2), illness (n = 2) or a major event in the participant's life on Day 2 (n = 1), and a lab closure between sessions due to COVID-19 (n = 1). One additional participant was tested but excluded post-hoc upon their disclosure of a psychiatric diagnosis, which was an exclusion criterion. The final sample size is thus N = 76. The experimental groups did not differ in gender composition (Pearson χ^2^ = .545, df = 2, *p* = .761), smoking status (Pearson χ^2^ = .554, df = 2, *p* = .758), use of hormonal contraceptives (Pearson χ^2^ = .042, df = 2, *p* = .979), BMI (F(2,75) = .478, *p* = .622, or age (F(2,75) = .251, *p* = .778 (see Table 1).

**Table 1:** Descriptive statistics.

		genetic feedback group			total	*p*
		high stress reactivity	low stress reactivity	low stress reactivity/diabetes		
Total (n)		26	25	25	76	
Gender (n)	male	11	13	11	35	.761
	female	15	12	14	41	
Smoking status (n)	yes	12	10	9	31	.758
Hormonal contraceptive use (female only, n)	yes	7	6	7	20	.979
Age (M, SD)		22.1 (2.6)	22.2 (2.4)	21.8 (2.2)	22.0 (2.4)	.778
Body mass index (M, SD)		22.8 (2.9)	22.2 (1.8)	22.2 (3.0)	22.4 (2.6)	.622

*Note. p*-values refer to group comparisons using one-way ANOVA (age, body mass index) or Pearson χ^2^-test.

### 3.2 Manipulation checks

#### 3.2.1 Deception

Most participants were successfully deceived into believing that a DNA sample had been taken and analyzed regarding their genetic predisposition for heightened stress reactivity. More precisely, 64 individuals (84.2%) agreed or strongly agreed with this statement, whereas four individuals each responded with do not agree, agree a little bit, and somewhat agree. An ANOVA with Welch correction for inhomogeneity (Levene test: *p* < .001) indicated no significant group differences between these ratings (*F*_Welch_(2,44.42) = 2.80, *p* = .071, *η*^*2*^ = .093).

Further supporting successful deception, individuals in the high stress feedback group agreed more strongly than the other groups that they believed they had a genetic predisposition to heightened stress reactivity (*F*_Welch_(2,42.97) = 26.95, *p* < .001, *η*^*2*^ = .549; Levene test: *p* < .001; high stress (*M* = 3.0, *SD* = 1.3) vs. low stress (*M* = 1.1, *SD* = .3): *p* < .001, *d* = 2.3; high stress vs. diabetes (*M* = 1.2, *SD* = .5): *p* < .001, *d* = 2.2, low stress vs. diabetes: *p* = .732). In contrast, the diabetes feedback group believed more strongly that they had a higher genetic risk for diabetes type 2 (Kruskal-Wallis H-test due to zero variance in the low stress group: *H(2)* = 54.91, *p* < .001, *η*^*2*^ = .667; diabetes (*M* = 3.7, *SD* = 1.2) vs high stress (*M* = 1.3, *SD* = .9): *p* < .001, diabetes vs. low stress (*M* = 1.0, *SD* = .0): *p* < .001, high vs. low stress: *p* = .152). Taken together, these results indicate that participants accepted the genetic feedback and that the manipulation successfully induced group-specific beliefs regarding genetic stress and diabetes risk.

#### 3.2.2 Stress induction

**Fig. 2:**
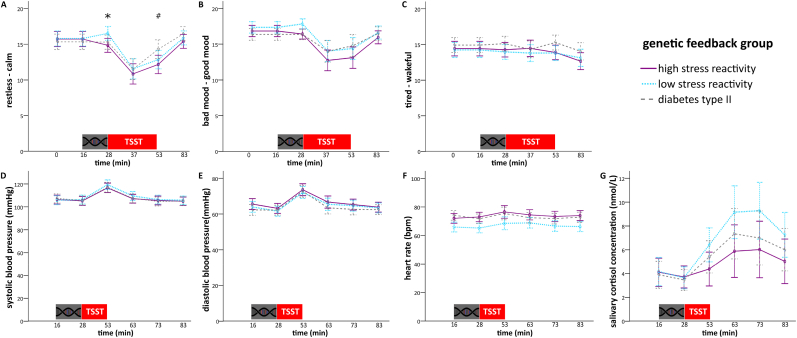
**Markers of stress reactivity for the three experimental groups on Day 2.** Top row shows subjective stress measures, i.e., **A** restlessness (n = 74), **B** subjective mood (n = 75), and **C** wakefulness (n = 76). Higher scores indicate calmness, better mood, and higher wakefulness, respectively. Bottom row shows physiological stress markers, i.e., **D** systolic blood pressure (n = 74), **E** diastolic blood pressure (n = 75), **F** heart rate (n = 76), and **G** salivary cortisol concentration (n = 62). Plots show means of raw data; error bars display 95%CI around the mean; time is indicated in minutes. Sampling time points for subjective stress markers (top row) and physiological stress markers (bottom row) differed as indicated by the colored blocks on the bottom of the graphs. Diverging sample sizes are due to missing data and removal of outliers defined by an absolute deviation of ≥3 SD from the mean of the full sample. *FB* feedback, *TSST* Trier Social Stress Test. ∗ indicates significantly stronger restlessness (i.e., lower scores) in the high stress feedback group compared to the low stress feedback group directly after feedback was provided. # indicates significantly stronger restlessness in the high stress feedback group directly after the TSST compared to the diabetes feedback group.

In line with abundant prior research, the TSST induced a pronounced subjective and physiological stress response (Fig. 2). More precisely, the TSST led to worse mood during the TSST and directly thereafter (main effect of time *F*(2.42,174.15) = 40.46, *p* < .001, *η*^2^_p_ = .360) and increased restlessness at the same time points (*F*(2.97,210.84) = 72.05, *p* < .001, *η*^*2*^_p_ = .504). Wakefulness was not affected by the TSST, as the significant main effect of time (*F*(3.39,247.3) = 7.72, *p* < .001, *η*^*2*^_p_ = .096) pertained only to a significant decline of wakefulness from time point 5 to 6 (*F*(1,73) = 17.86, *p* < .001) as indicated by repeated contrasts (all other *p* > .05). Regarding cardiovascular measures, the TSST also induced a pronounced increase from before to directly after the TSST in diastolic blood pressure (main effect of time *F*(4.10,295.42) = 67.02, *p* < .001, *η*^*2*^_p_ = .482), systolic blood pressure (main effect of time *F*(4.13,293.45) = 88.31, *p* < .001, *η*^*2*^_p_ = .554), and heart rate (main effect of time *F*(3.68,268.37) = 7.27, *p* < .001, *η*^*2*^_*p*_ = .091). Finally, the TSST also induced a pronounced response in log-transformed cortisol concentrations (main effect of time *F*(1.42,83.82) = 27.65, *p* < .001 *η*^*2*^_*p*_ = .319), as cortisol concentrations declined until timepoint 2, then rose until timepoint 4, and fell again from timepoint 5 to timepoint 6 (all *p* < .001).

### 3.3 Effects of genetic risk feedback

#### 3.3.1 Self-reported effects

Supporting effects of bogus genetic feedback on subjective experience, the high-stress feedback group reported being more concerned by the purported genetic test result at the end of the experiment than participants in the other groups (*F*_*Welch*_(2,48.18) = 11.65, *p* < .001, *η*^*2*^ = .262). Post-hoc comparisons showed higher concern in the high-stress group (M = 2.8, SD = 1.3) compared to both the low-stress group (M = 1.4, SD = .9, *p* < .001, *d* = 1.35) and the diabetes control group (M = 1.9, SD = 1.0, *p* = .002, *d* = .859) with large effects, whereas the latter two groups did not differ significantly (*p* = .087). Additionally, the three groups differed in self-reported restlessness over the course of the experiment as indicated by a significant time×group interaction (*F*(5.94,210.84) = 2.38, *p* = .031, *η*^*2*^_p_ = .063). More precisely, participants who were suggested to be stress susceptible reported higher restlessness, i.e., more subjective stress, directly after receiving this information compared to the low-stress feedback group (*T*(49) = −2.47, *p* = .017, *d* = −.692), whereas we found no difference between the two control groups (*T*(47) = 1.90, *p* = .064). Additionally, participants in the high stress reactivity feedback group were more restless than the diabetes control group directly after the TSST (*T*(44.95) = −2.49, *p* = .017, *d* = −.694), possibly indicating delayed subjective recovery. In contrast, we found no difference compared to the low-stress control group (*T*(49) = −.78, *p* = .439) or between the two control groups (*T*(47) = −1.71, *p* = .094). Genetic feedback did not affect restlessness at other timepoints, self-reported mood (time×group interaction *F*(4.84,174.15) = 1.47, *p* = .205, *η*^*2*^_p_ = .039), or wakefulness (time×group interaction *F*(6.78,247.30) = 1.15, *p* = .325, *η*^*2*^_p_ = .030).

#### 3.3.2 Effects on physiological stress reactivity

In contrast to its effects on feeling more concerned and restless, genetic feedback did not affect physiological stress reactivity. More precisely, we found no time×group interaction on blood pressure (diastolic: *F*(8.21,295,42) = .79, *p* = .615, systolic: *F*(8.27,293.45) = .81, *p* = .601), heart rate (*F*(7.35,268.37) = 1.46, *p* = .178), or log-transformed cortisol concentration (*F*(2.84,83.82) = 1.736, *p* = .169). There was a main effect of group specifically for heart rate (*F*(2,73) = 4.93, *p* = .010, *η*^*2*^_p_ = .119, low stress < high stress: *p* = .005, low stress < diabetes: *p* = .014), but this difference was independent of time and therefore cannot be attributed to our feedback manipulation. These results did not change when gender was included as a covariate for cortisol concentrations or when stress reactivity scores (baseline-to-peak for cardiovascular measures or AUC_i/g_ for cortisol, respectively) were analyzed.

#### 3.3.3 Effects on retrospectively reported chronic stress

To assess whether the information to carry a genetic predisposition for heightened stress reactivity affected self-reported retrospective evaluations of chronic stress, we analyzed whether the TCSS scores changed from Day 1 to Day 2. Contrary to our expectation, the TCSS scores were not affected by group, time, or their interaction (main effect of group: *F*(2,73) = .79, *p* = .456, *η*^*2*^_p_ = .021; main effect of time: *F*(1,73) = 1.43, *p* = .236, *η*^*2*^_p_ = .019; group×time interaction: *F*(2,73) = 1.91, *p* = .156, *η*^*2*^_p_ = .050).

#### 3.3.4 Effects on cognitive impairments

We observed medium to large decreases in all indices of backward digit span from before to after the TSST (Table 2, main effects of time: largest span: *F*(1,73) = 9.69, *p* = .003, *η*^*2*^_p_ = .117; number of correct trials: *F*(1,72) = 31.34, *p* < .001, *η*^*2*^_p_ = .303; product: *F*(1,72) = 18.50, *p* < .001, *η*^*2*^_p_ = .204). However, this effect was not modulated by genetic risk feedback (largest span: *F*(2,73) = .65, *p* = .527; number of correct trials: *F*(2,72) = .74, *p* = .481; product: *F*(2,72) = .60, *p* = .550). In contrast, forward digit span did not change over the course of the experiment (all *F*(1,73) < 1, *p* > .05), and again, there was no evidence for a group×time interaction (largest span and number of correct trials: *F*(2,73) < 1, *p* > .05, product: *F*(2,71) < 1, *p* > .05).

**Table 2:** Digit span measures for all experimental groups at baseline and after stress induction.

		genetic feedback group					
		high stress reactivity		low stress reactivity		low stress reactivity/diabetes	
		Mean	SD	Mean	SD	Mean	SD
*forward condition*							
largest correct span	baseline	5.54	1.12	5.16	1.09	5.56	1.03
	post stress	5.77	1.02	5.16	1.01	5.60	1.17
number of correct trials	baseline	9.88	1.85	9.36	2.08	10.04	1.83
	post stress	10.27	1.59	9.44	1.68	10.04	1.85
product	baseline	56.62	21.96	50.32	19.08	57.60	21.83
	post stress	60.69	19.20	50.28	17.92	58.20	22.29

*backward condition*							
largest correct span	baseline	4.69	.95	4.92	1.10	4.60	1.36
	post stress	4.50	.93	4.48	.90	4.08	1.17
number of correct trials	baseline	8.62	1.76	8.80	1.88	8.60	2.49
	post stress	7.85	1.77	7.68	1.60	7.20	2.16
product	baseline	42.00	15.81	45.24	19.18	42.84	25.49
	post stress	36.77	14.56	35.64	13.20	31.72	16.89

*Note.* Product refers to the product of largest digit span and number of correct trials per condition.

## 4 Discussion

In this study, we set out to test whether bogus feedback about an individual's genetic susceptibility to stress would induce subjective, physiological, or cognitive effects in line with the expectation raised by the genetic feedback. Using an integrated approach assessing various stress-related psychological and physiological measures, we found that an alleged genetic susceptibility to stress induced small increases in restlessness and larger increases in concern compared to both control groups with supposedly low stress susceptibility. Moreover, after a standardized laboratory stressor, subjective recovery appeared delayed regarding restlessness in the high-stress feedback group. In contrast, while physiological measures of stress did show the expected stress response, we found no evidence that genetic feedback modulated its magnitude. Likewise, we replicated a frequently reported adverse cognitive effect after stress, i.e., reduced WM, but again no effect of genetic feedback on this impairment. To summarize, our findings from self-reports demonstrate that merely believing to be genetically susceptible to stress can alter subjective responses to stress and delay recovery following a stressful encounter.

Our results can be interpreted in light of several theories regarding how expectations can affect human experience, behavior, and physiology. For instance, Rief and Petrie [13] proposed psychological expectations as a central mechanism underlying placebo and nocebo effects. Importantly, the authors argue that the strength of an expectation depends on how well it has been learned, which in turn is influenced by individual learning rates and how frequently the expectation was confirmed in the past. This highlights the role of pre-existing expectations prior to our manipulation and may explain the observed distinction between subjective and physiological or cognitive effects. As stress is a common everyday experience, participants likely entered the experiment with pre-existing beliefs regarding their own stress susceptibility. Thus, a single verbal feedback intervention may have only a limited immediate effect but could shape future experiences with stressors over time. In line with this reasoning, the impaired subjective recovery observed in the high stress susceptibility group could contribute to stronger affective, and potentially also physiological or cognitive, effects when encountering subsequent stressors. Over longer time frames, genetic feedback may become integrated into existing belief structures and thereby increasingly influence stress appraisal. For instance, we found effects on restlessness, a scale ranging from feeling tense, aroused, and nervous to feeling calm and composed, but not on mood, which reflects more general affective valence. This pattern suggests that the genetic feedback primarily affected self-reported arousal and perceived stress-related tension, which are more directly captured by the restlessness dimension than by global mood ratings. However, longer time-frames may induce broader affective effects, or extend to physiological and cognitive changes under stress. It should be noted, however, that ethical considerations limit the use of extended time frames in deception studies. One study without deception employed an 8-months interval between communicating genetic risk for Alzheimer's disease and memory testing and reported pronounced cognitive effects [10], suggesting that the effects of genetic feedback may unfold over time.

In addition to expectation-based mechanisms, other factors may influence how individuals interpret genetic feedback, such as confirmation bias. A qualitative study suggested that individuals receiving genetic feedback often do not use this information to update prior beliefs or guide lifestyle changes [44]. Instead, genetic information may reinforce pre-existing beliefs about personal risk or, when inconsistent with prior beliefs, lead individuals to question the credibility of the information itself. Taken together, both expectation models and confirmation bias highlight the importance of assessing prior beliefs before providing genetic feedback in future studies, as these beliefs may moderate the psychological impact of risk communication.

Another theoretical framework on how expectation may shape stress reactivity was proposed by De Raedt and Hooley [45]. The neurocognitive framework for regulation expectation (NFRE) emphasizes the role of expectations regarding one's own regulatory abilities during stress anticipation. According to this framework, individuals who expect to cope successfully with a stressor may engage in proactive regulation during anticipation, thereby facilitating regulation of the stress response once the stressor occurs. This perspective aligns with the appraisal model by Lazarus and Folkman [19], which posits that both the evaluation of a situation as threatening (primary appraisal) and the perceived ability to cope with it (secondary appraisal) critically determine stress responses. Accordingly, extending the anticipation period and increasing the sampling frequency of stress markers may provide further insight into the dynamic effects of genetic feedback. Supporting this notion, Engert et al. [46] demonstrated naturally occurring anticipatory cortisol responses to the TSST in some individuals, peaking during stress exposure, a phase during which saliva sampling is often avoided to prevent interference. Continuous assessment of stress markers may therefore represent a useful avenue for future research.

Contrary to our second hypothesis, feedback about participants’ genetic susceptibility to stress did not affect retrospective reports of chronic stress. Although not directly comparable, this finding differs from a prior study showing that genetic feedback can affect retrospective assessments of depressive symptoms [11]. One possible explanation is the short time interval between feedback and chronic stress assessment in our study. Alternatively, the TICS may not represent an optimal measure for detecting such effects. Recent work suggests that TICS scores reflect not only variance in chronic stress levels but are also influenced by factors not directly related to stress, e. g., fatigue and neuroticism [47], which may be more stable over time.

Regarding our third question on cognitive functioning, we focused on WM as one specific cognitive domain, which was frequently, though not always consistently, reported to be impaired following acute stress induction (e.g., Ref. [28,48]). Acute stress affects a broad range of cognitive functions, including other forms of memory, executive functions, decision making, and behavior (e.g. Ref. [[49], [50], [51], [52], [53], [54]]). Consistent with meta-analytic findings [28], we found impairments after the TSST particularly in the backward condition, supporting previous evidence of stronger stress-induced WM impairments under higher WM load compared to lower load (i.e., forward condition). A greater involvement of executive processes in the backward condition compared to the forward condition has been assumed, whereas the latter primarily reflects passive storage or maintenance of information [48,55]. However, in the absence of a non-stressed control group, these changes cannot be unequivocally attributed to stress and may also reflect time-dependent factors such as fatigue or task repetition. More importantly, we found no evidence that the changes after the TSST were influenced by genetic feedback, which may again relate to the short time frame of our study, as discussed above.

One strength of our study is that we tested whether possible effects of (bogus) genetic information were specifically related to the information provided (i.e., heightened stress susceptibility) rather than merely reflecting the valence of the information (good vs. bad news). To our knowledge, the inclusion of a second “bad news” control group has not been implemented in prior studies but helps to distinguish specific from unspecific effects of individualized risk feedback. However, it could be argued that this bad news condition (i.e., increased risk for type 2 diabetes) may not have been sufficiently adverse, as it did not induce concern or changes in subjective stress levels. Careful consideration of appropriate control conditions therefore remains important for future research on individualized feedback. One limitation of the present study is that the sample consisted mainly of psychology students, who may possess greater knowledge about genetic influences on interindividual differences than the general population. This may attenuate effects of genetic feedback, as individuals with higher numeracy and better understanding of polygenic risk scores have been shown to exhibit less negative emotional responses to genetic risk feedback [9]. Accordingly, our results may represent a conservative estimation of the effects of genetic feedback. Future studies could address this issue using moderation analyses, although substantially larger sample sizes would be required. Moreover, we used a between-subjects design to investigate the effects of genetic feedback on stress-related variables, whereas pre-post designs (e.g., Ref. [17]) may be more sensitive to detect changes. However, in stress-research such designs are more difficult to implement, as repeated stress exposure leads to habituation [56], likely due to reduced unpredictability. Additionally, we used a German self-report form to assess subjective stress, which is frequently used in German-speaking countries but less well-known internationally, potentially limiting comparability with other studies. Moreover, hormonal contraceptive use may have influenced cortisol responses; however, this factor was accounted for as a covariate while allowing for a more representative sample.

Finally, it should be emphasized that we focused our research on the effects of genetic feedback on the individual receiving this information. However, concerns have been raised about possible negative consequences of DTC genetic testing beyond these direct effects. For instance, DTC genetic tests may pose a high burden on health services and have a potential for unnecessary harm, particularly given that the false-positive rates were sometimes found to be high [57]. This could be further exacerbated by the fact that DTC genetic tests inherently also provide information relevant to individuals that are genetically related to test takers.

To summarize, DTC genetic testing is becoming increasingly common, yet its psychological and behavioral consequences remain incompletely understood. The present findings, although of small effect size (particularly for restlessness), suggest that genetic risk feedback may primarily influence self-reported arousal and perceived stress-related tension rather than broader mood or physiological stress responses. This pattern is consistent with the idea that beliefs about biological vulnerability may shape specific aspects of subjective stress experience, even in the absence of measurable physiological changes. Such effects are likely to depend on the specific condition addressed, prior experiences of the individual, and how genetic feedback is interpreted. Moreover, the consequences of genetic feedback may unfold over time, highlighting the importance of longitudinal assessments of feedback effects. Together, our findings underscore the need for further research on the impact of individualized genetic feedback.

## CRediT authorship contribution statement

**Susanne Vogel:** Conceptualization, Data curation, Formal analysis, Investigation, Methodology, Project administration, Validation, Visualization, Writing – original draft. **Marie-Christin Barthel:** Investigation, Project administration, Writing – review & editing. **Kim Lars Fricke:** Conceptualization, Methodology, Writing – original draft. **Markus Muehlhan:** Conceptualization, Writing – review & editing. **Nina Alexander:** Conceptualization, Resources, Writing – review & editing.

## Declaration of competing interest

The authors declare that they have no known competing financial interests or personal relationships that could have appeared to influence the work reported in this paper.
