# Supplementary material for: Effects of bogus genetic feedback on subjective and physiological responses to acute stress
Source: Compr Psychoneuroendocrinol. 2026 Jun 25;27:100358. doi: 10.1016/j.cpnec.2026.100358 (PMC13329565; doi:10.1016/j.cpnec.2026.100358)
Supplement: Multimedia component 1 [file mmc1.docx]

**Supplementary material**

The following pages contain the original material used to provide bogus genetic feedback, depending on group assignment: Feedback for the experimental group (receiving high genetic risk information) can be found on p. 2, feedback for the low-risk control group on p. 4, and feedback for the “bad news” control group on pages 6-7. Additionally, participants also received a sequencing chromatogram, purportedly reflecting the DNA test performed (p. 10).

Please note that all material is in German as the study was conducted in German-speaking individuals. English translations are supplied per condition after the German version for transparency and to aid readers’ understanding of the information presented to participants (i.e., pages 3, 5, and 8-9).

**DNA-ARRAY DIAGNOSTIK**

Name: **[…]** Datum Probennahme: **[XX/XX/XXXX]**

Proben-Nummer: DXP9 Datum Labor Analyse: **[XX/XX/XXXX]**

Probenart: PyroMark Assay

**TESTPRINZIP**

Mit Hilfe moderner DNA-Analyseverfahren lassen sich Risikogenvarianten bestimmen, die mit bestimmten menschlichen Eigenschaften und Erkrankungen zusammenhängen. Innerhalb dieser Studie interessieren wir uns für eine Kombination von Risikogenvarianten, mit denen wir zuverlässig vorhersagen können, ob eine Person eine besonders starke Stressanfälligkeit zeigt (z.B. setzen Träger dieser Risikogenvarianten mehr Stresshormone frei und zeigen einen stärkeren Herzratenanstieg unter Stress). Wir analysieren zu diesem Zweck 5 Risikogenvarianten innerhalb der Gene MAOA, SLC6A4, FKBP5, NR3C1 und NR3C2, mit deren Hilfe in vorangegangenen Studien das Risiko für eine erhöhte Stressanfälligkeit verlässlich vorhergesagt werden konnte, um ein individuelles Risikoprofil zu erstellen. Dabei wird pro Gen ein Marker untersucht, bei dem bekannt ist, dass eine Variation mit einem erhöhten Risiko für stärkere Stressreaktionen einhergeht („Risiko-Allel“). Da Sie immer eine Genvariante von Ihrem Vater und eine von Ihrer Mutter geerbt haben, können Sie pro Genort entweder 0, 1 oder 2 Risikogenvarianten tragen. Ihr individuelles Risikoprofil ergibt sich aus der Anzahl aller Risikogenvarianten an den 5 analysierten Genorten und kann somit einen Wert von 0 (sehr geringes Risiko) bis 10 (sehr hohes Risiko) annehmen. Zusätzlich können wir mit Hilfe dieses genetischen Profils auch das Risiko für einige gängige chronische Erkrankungen vorhersagen. Sollten wir im Zuge unserer Analysen zufällig auf ein genetisch bedingtes, erhöhtes Risiko für chronische Erkrankungen stoßen, werden wir Ihnen dies zusätzlich zurückmelden.

Ergebnis der DNA-Analyse der getesteten genetischen Marker

| **Marker** | **Risiko-Allel** | **Persönlicher Genotyp** |
| --- | --- | --- |
| rs6323 (MAOA) | [G] | [G;G] |
| rs25531 (SLC6A4) | [A] | [G;A] |
| rs1360780 (FKBP5) | [ T] | [T;T] |
| rs10482605 (NR3C1) | [C] | [C;C] |
| rs5522 (NR3C2) | [A] | [A;A] |

Testparameter: Sensitivität 99.1%, Spezifität 99.6% und positiver prädiktiver Wert 94.8% (Monsuur et al, 2008). Methode: Taqman® Real-Time PCR

**TESTERGEBNISSE**


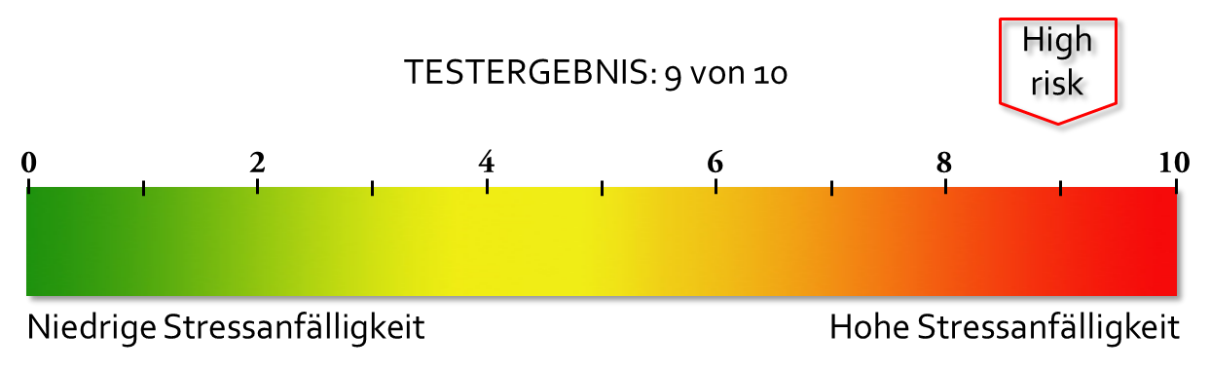
Stressanfälligkeit

Das Ergebnis des DNA Test indiziert ein hohes Risiko für eine starke Stressanfälligkeit. Dies bedeutet z. B., dass Träger dieses genetischen Risikoprofils mehr Stresshormone freisetzen und einen stärkeren Herzratenanstieg unter Stress zeigen.

Sonstige Testergebnisse

--- keine ---

**English translation:**

**DNA-ARRAY ANALYSIS**

Name: **[…]** Date of sample collection: **[XX/XX/XXXX]**

Sample number: DXP9   Date of analysis: **[XX/XX/XXXX]**

Sample type: PyroMark Assay

**TEST PRINCIPLE**

Using modern DNA analysis techniques, it is possible to identify genetic risk variants that are associated with certain human traits and diseases. In the present study, we are interested in a combination of genetic risk variants that allows us to reliably predict whether a person shows particularly high stress sensitivity (e.g., carriers of these genetic risk variants release higher levels of stress hormones and show a stronger increase in heart rate under stress). For this purpose, we analyze 5 genetic risk variants within the genes MAOA, SLC6A4, FKBP5, NR3C1, and NR3C2, which previous studies have shown to reliably predict an increased risk of heightened stress sensitivity, in order to create an individual risk profile. To do so, one marker is examined for each gene, for which it is known that a specific variant is associated with an increased risk of stronger stress responses (“risk allele”). Because you inherit one genetic variant from your father and one from your mother, you may carry 0, 1, or 2 risk variants per gene locus. Your individual risk profile is based on the total number of risk variants across the 5 analyzed gene loci and can therefore range from 0 (very low risk) to 10 (very high risk). In addition, this genetic profile can also be used to predict the risk for some common chronic diseases. Should our analyses incidentally reveal a genetically determined increased risk for chronic diseases, we will provide you with this information as well.

Results of the DNA analysis of the tested genetic markers

| **marker** | **risk allele** | **individual genotype** |
| --- | --- | --- |
| rs6323 (MAOA) | [G] | [G;G] |
| rs25531 (SLC6A4) | [A] | [G;A] |
| rs1360780 (FKBP5) | [ T] | [T;T] |
| rs10482605 (NR3C1) | [C] | [C;C] |
| rs5522 (NR3C2) | [A] | [A;A] |

Test parameters: sensitivity 99.1%, specificity 99.6%, positive predictive value 94.8% (Monsuur et al., 2008). Method: Taqman® real-time PCR


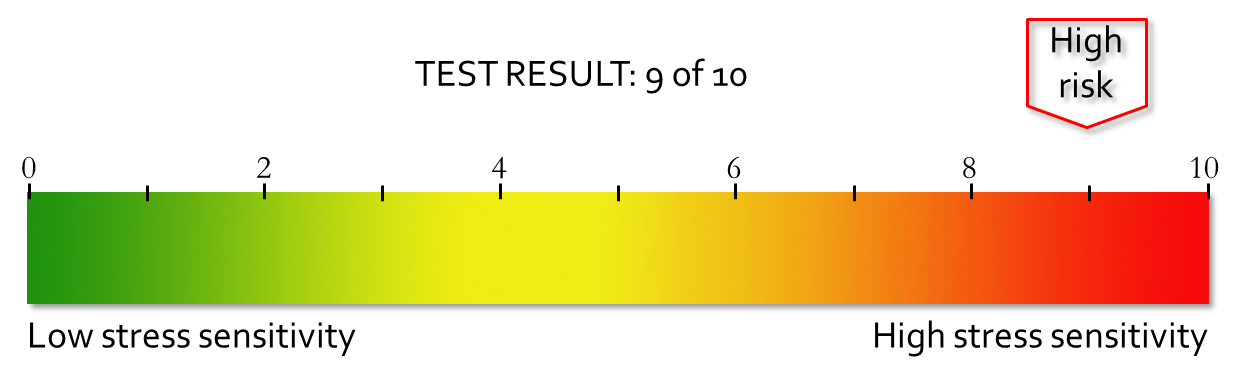
**TEST RESULTS**

Stress sensitivity

The result of the DNA test indicates a high risk of being very susceptible to stress. This means, for instance, that carriers of this genetic risk profile release more stress hormones and show a greater heart rate increase during stress.

Other test results

— none

**DNA-ARRAY DIAGNOSTIK**

Name: **[…]** Datum Probennahme: **[XX/XX/XXXX]**

Proben-Nummer: DXP9 Datum Labor Analyse: **[XX/XX/XXXX]**

Probenart: PyroMark Assay

TESTPRINZIP

Mit Hilfe moderner DNA-Analyseverfahren lassen sich Risikogenvarianten bestimmen, die mit bestimmten menschlichen Eigenschaften und Erkrankungen zusammenhängen. Innerhalb dieser Studie interessieren wir uns für eine Kombination von Risikogenvarianten, mit denen wir zuverlässig vorhersagen können, ob eine Person eine besonders starke Stressanfälligkeit zeigt (z.B. setzen Träger dieser Risikogenvarianten mehr Stresshormone frei und zeigen einen stärkeren Herzratenanstieg unter Stress). Wir analysieren zu diesem Zweck 5 Risikogenvarianten innerhalb der Gene MAOA, SLC6A4, FKBP5, NR3C1 und NR3C2, mit deren Hilfe in vorangegangenen Studien das Risiko für eine erhöhte Stressanfälligkeit verlässlich vorhergesagt werden konnte, um ein individuelles Risikoprofil zu erstellen. Dabei wird pro Gen ein Marker untersucht, bei dem bekannt ist, dass eine Variation mit einem erhöhten Risiko für stärkere Stressreaktionen einhergeht („Risiko-Allel“). Da Sie immer eine Genvariante von Ihrem Vater und eine von Ihrer Mutter geerbt haben, können Sie pro Genort entweder 0, 1 oder 2 Risikogenvarianten tragen. Ihr individuelles Risikoprofil ergibt sich aus der Anzahl aller Risikogenvarianten an den 5 analysierten Genorten und kann somit einen Wert von 0 (sehr geringes Risiko) bis 10 (sehr hohes Risiko) annehmen. Zusätzlich können wir mit Hilfe dieses genetischen Profils auch das Risiko für einige gängige chronische Erkrankungen vorhersagen. Sollten wir im Zuge unserer Analysen zufällig auf ein genetisch bedingtes, erhöhtes Risiko für chronische Erkrankungen stoßen, werden wir Ihnen dies zusätzlich zurückmelden.

Ergebnis der DNA-Analyse der getesteten genetischen Marker

| **Marker** | **Risiko-Allel** | **Persönlicher Genotyp** |
| --- | --- | --- |
| rs6323 (MAOA) | [G] | [T;T] |
| rs25531 (SLC6A4) | [A] | [A;G] |
| rs1360780 (FKBP5) | [ T] | [C;T] |
| rs10482605 (NR3C1) | [C] | [T;T] |
| rs5522 (NR3C2) | [A] | [G;G] |

Testparameter: Sensitivität 99.1%, Spezifität 99.6% und positiver prädiktiver Wert 94.8% (Monsuur et al, 2008). Methode: Taqman® Real-Time PCR

TESTERGEBNISSE


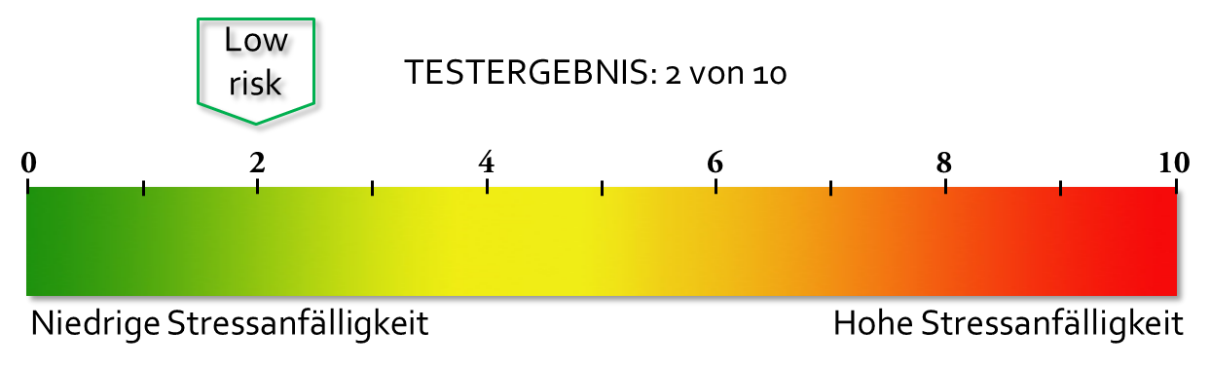
Stressanfälligkeit

Das Ergebnis des DNA Test indiziert ein niedriges Risiko für eine starke Stressanfälligkeit. Dies bedeutet z.B., dass Träger dieses genetischen Risikoprofils weniger Stresshormone freisetzen und einen geringeren Herzratenanstieg unter Stress zeigen.

Sonstige Testergebnisse

--- keine ---

**English translation:**

**DNA-ARRAY ANALYSIS**

Name: **[…]** Date of sample collection: **[XX/XX/XXXX]**

Sample number: DXP9   Date of analysis: **[XX/XX/XXXX]**

Sample type: PyroMark Assay

**TEST PRINCIPLE**

Using modern DNA analysis techniques, it is possible to identify genetic risk variants that are associated with certain human traits and diseases. In the present study, we are interested in a combination of genetic risk variants that allows us to reliably predict whether a person shows particularly high stress sensitivity (e.g., carriers of these genetic risk variants release higher levels of stress hormones and show a stronger increase in heart rate under stress). For this purpose, we analyze 5 genetic risk variants within the genes MAOA, SLC6A4, FKBP5, NR3C1, and NR3C2, which previous studies have shown to reliably predict an increased risk of heightened stress sensitivity, in order to create an individual risk profile. To do so, one marker is examined for each gene, for which it is known that a specific variant is associated with an increased risk of stronger stress responses (“risk allele”). Because you inherit one genetic variant from your father and one from your mother, you may carry 0, 1, or 2 risk variants per gene locus. Your individual risk profile is based on the total number of risk variants across the 5 analyzed gene loci and can therefore range from 0 (very low risk) to 10 (very high risk). In addition, this genetic profile can also be used to predict the risk for some common chronic diseases. Should our analyses incidentally reveal a genetically determined increased risk for chronic diseases, we will provide you with this information as well.

Results of the DNA analysis of the tested genetic markers

| **marker** | **risk allele** | **individual genotype** |
| --- | --- | --- |
| rs6323 (MAOA) | [G] | [T;T] |
| rs25531 (SLC6A4) | [A] | [A;G] |
| rs1360780 (FKBP5) | [ T] | [C;T] |
| rs10482605 (NR3C1) | [C] | [T;T] |
| rs5522 (NR3C2) | [A] | [G;G] |

Test parameters: sensitivity 99.1%, specificity 99.6%, positive predictive value 94.8% (Monsuur et al., 2008). Method: Taqman® real-time PCR


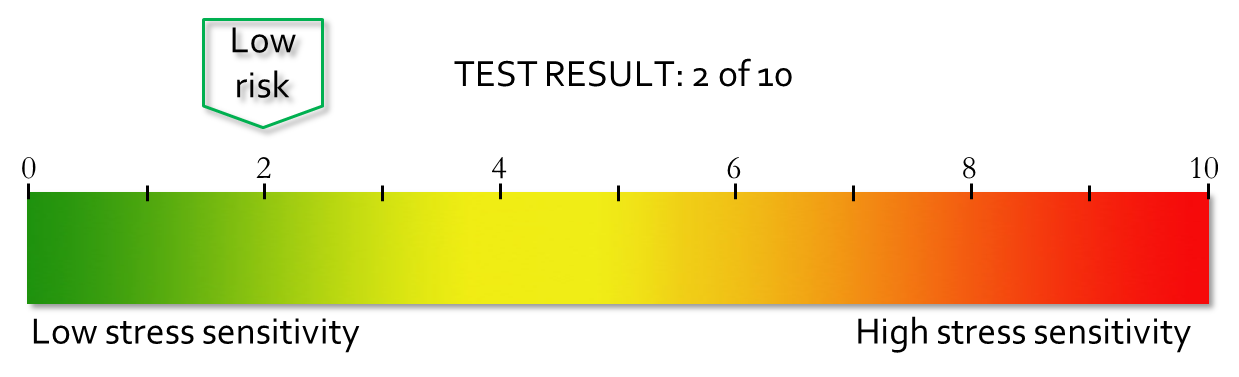
**TEST RESULTS**

Stress sensitivity

The result of the DNA test indicates a low risk of being susceptible to stress. This means, for example, that carriers of this genetic risk profile release lower levels of stress hormones and show a smaller increase in heart rate during stress.

Other test results

— none

**DNA-ARRAY DIAGNOSTIK**

Name: **[…]** Datum Probennahme: **[XX/XX/XXXX]**

Proben-Nummer: DXP9 Datum Labor Analyse: **[XX/XX/XXXX]**

Probenart: PyroMark Assay

TESTPRINZIP

Mit Hilfe moderner DNA-Analyseverfahren lassen sich Risikogenvarianten bestimmen, die mit bestimmten menschlichen Eigenschaften und Erkrankungen zusammenhängen. Innerhalb dieser Studie interessieren wir uns für eine Kombination von Risikogenvarianten, mit denen wir zuverlässig vorhersagen können, ob eine Person eine besonders starke Stressanfälligkeit zeigt (z.B. setzen Träger dieser Risikogenvarianten mehr Stresshormone frei und zeigen einen stärkeren Herzratenanstieg unter Stress). Wir analysieren zu diesem Zweck 5 Risikogenvarianten innerhalb der Gene MAOA, SLC6A4, FKBP5, NR3C1 und NR3C2, mit deren Hilfe in vorangegangenen Studien das Risiko für eine erhöhte Stressanfälligkeit verlässlich vorhergesagt werden konnte, um ein individuelles Risikoprofil zu erstellen. Dabei wird pro Gen ein Marker untersucht, bei dem bekannt ist, dass eine Variation mit einem erhöhten Risiko für stärkere Stressreaktionen einhergeht („Risiko-Allel“). Da Sie immer eine Genvariante von Ihrem Vater und eine von Ihrer Mutter geerbt haben, können Sie pro Genort entweder 0, 1 oder 2 Risikogenvarianten tragen. Ihr individuelles Risikoprofil ergibt sich aus der Anzahl aller Risikogenvarianten an den 5 analysierten Genorten und kann somit einen Wert von 0 (sehr geringes Risiko) bis 10 (sehr hohes Risiko) annehmen. Zusätzlich können wir mit Hilfe dieses genetischen Profils auch das Risiko für einige gängige chronische Erkrankungen vorhersagen. Sollten wir im Zuge unserer Analysen zufällig auf ein genetisch bedingtes, erhöhtes Risiko für chronische Erkrankungen stoßen, werden wir Ihnen dies zusätzlich zurückmelden.

Ergebnis der DNA-Analyse der getesteten genetischen Marker

| **Marker** | **Risiko-Allel** | **Persönlicher Genotyp** |
| --- | --- | --- |
| rs6323 (MAOA) | [G] | [T;T] |
| rs25531 (SLC6A4) | [A] | [G;G] |
| rs1360780 (FKBP5) | [ T] | [C;C] |
| rs10482605 (NR3C1) | [C] | [C;C] |
| rs5522 (NR3C2) | [A] | [G;G] |

Testparameter: Sensitivität 99.1%, Spezifität 99.6% und positiver prädiktiver Wert 94.8% (Monsuur et al, 2008). Methode: Taqman® Real-Time PCR

TESTERGEBNISSE


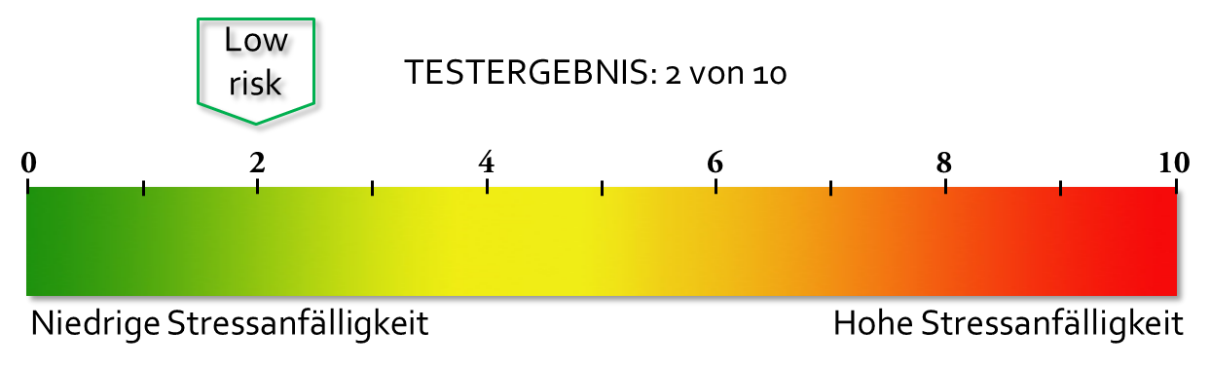
Stressanfälligkeit

Das Ergebnis des DNA Test indiziert ein niedriges Risiko für eine starke Stressanfälligkeit. Dies bedeutet z.B., dass Träger dieses genetischen Risikoprofils weniger Stresshormone freisetzen und einen geringeren Herzratenanstieg unter Stress zeigen.

Sonstige Testergebnisse

Das Ergebnis des DNA Test indiziert ein hohes Risiko für Diabetes mellitus Typ-II, sogenannter „Altersdiabetes“. Ein hohes Risiko für Diabetes Typ-II führt aufgrund einer sich stetig entwickelnden Insulinresistenz zu erhöhten Blutzuckerwerten. Dies bedeutet, dass Sie insbesondere mit zunehmendem Alter eine spezifische (z.B. zuckerarme) Diät einhalten sollten.

**English translation:**

**DNA-ARRAY ANALSYIS**

Name: **[…]** Date of sample collection: **[XX/XX/XXXX]**

Sample number: DXP9   Date of analysis: **[XX/XX/XXXX]**

Sample type: PyroMark Assay

TEST PRINCIPLE

Using modern DNA analysis techniques, it is possible to identify genetic risk variants that are associated with certain human traits and diseases. In the present study, we are interested in a combination of genetic risk variants that allows us to reliably predict whether a person shows particularly high stress sensitivity (e.g., carriers of these genetic risk variants release higher levels of stress hormones and show a stronger increase in heart rate under stress). For this purpose, we analyze 5 genetic risk variants within the genes MAOA, SLC6A4, FKBP5, NR3C1, and NR3C2, which previous studies have shown to reliably predict an increased risk of heightened stress sensitivity, in order to create an individual risk profile. To do so, one marker is examined for each gene, for which it is known that a specific variant is associated with an increased risk of stronger stress responses (“risk allele”). Because you inherit one genetic variant from your father and one from your mother, you may carry 0, 1, or 2 risk variants per gene locus. Your individual risk profile is based on the total number of risk variants across the 5 analyzed gene loci and can therefore range from 0 (very low risk) to 10 (very high risk). In addition, this genetic profile can also be used to predict the risk for some common chronic diseases. Should our analyses incidentally reveal a genetically determined increased risk for chronic diseases, we will provide you with this information as well.

Results of the DNA analysis of the tested genetic markers

| **marker** | **risk allele** | **individual genotype** |
| --- | --- | --- |
| rs6323 (MAOA) | [G] | [T;T] |
| rs25531 (SLC6A4) | [A] | [A;G] |
| rs1360780 (FKBP5) | [ T] | [C;T] |
| rs10482605 (NR3C1) | [C] | [T;T] |
| rs5522 (NR3C2) | [A] | [G;G] |

Test parameters: sensitivity 99.1%, specificity 99.6%, positive predictive value 94.8% (Monsuur et al., 2008). Method: Taqman® real-time PCR

TEST RESULTS


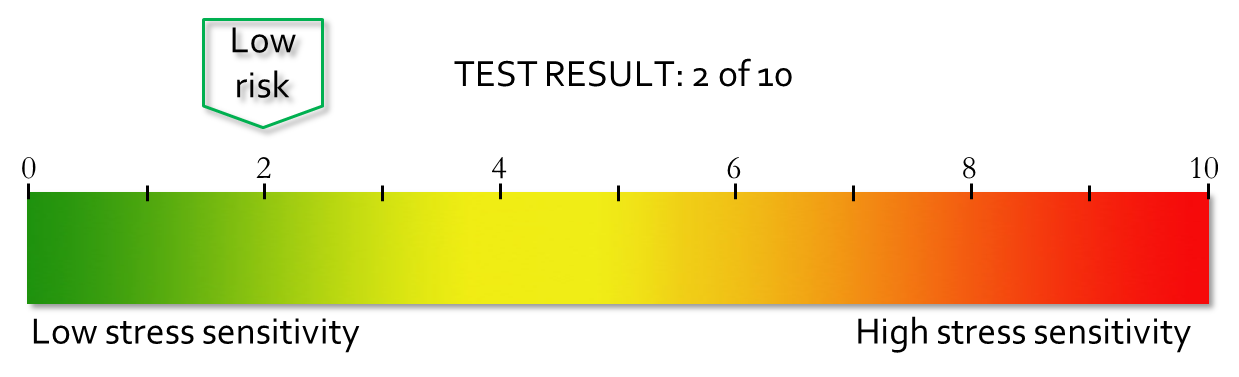
Stress sensitivity

The result of the DNA test indicates a low risk of being susceptible to stress. This means, for example, that carriers of this genetic risk profile release lower levels of stress hormones and show a smaller increase in heart rate during stress.

Other test results

The result of the DNA test indicates a high risk of type II diabetes mellitus, so-called “adult-onset diabetes”. A high risk of type II diabetes leads to increased blood glucose levels due to a steadily developing insulin resistance. This means that you should follow a specific (e.g., low-sugar) diet, especially as you get older.

Well: A14

Assay: NR3CI-DD_P1_S3new5 Sample ID: DXP9

Sequence to analyze: TYGYGTYGTYGTYGTYGGGTYGAGTTGYGTGAAGTGTGTTATTTYGAAAGGGGTTAYGGGGTTGTAYGGAAAYGGTGTYG

Note: rec+1
